# StreAM-$T_g$: algorithms for analyzing coarse grained RNA dynamics based on Markov models of connectivity-graphs

**DOI:** 10.1186/s13015-017-0105-0

**Published:** 2017-05-30

**Authors:** Sven Jager, Benjamin Schiller, Philipp Babel, Malte Blumenroth, Thorsten Strufe, Kay Hamacher

**Affiliations:** 1 Department of Biology, TU Darmstadt, Schnittspahnstr. 2, 64283 Darmstadt, Germany; 2 Department of Computer Science, TU Dresden, Nöthnitzer Str. 46, 01187 Dresden, Germany; 3 Department of Biology, Department of Computer Science, Department of Physics, TU Darmstadt, Schnittspahnstr. 2, 64283 Darmstadt, Germany

**Keywords:** RNA, Markovian dynamics, Dynamic graphs, Molecular dynamics, Coarse graining, Synthetic biology

## Abstract

**Background:**

In this work, we present a new coarse grained representation of RNA dynamics. It is based on adjacency matrices and their interactions patterns obtained from molecular dynamics simulations. RNA molecules are well-suited for this representation due to their composition which is mainly modular and assessable by the secondary structure alone. These interactions can be represented as adjacency matrices of *k* nucleotides. Based on those, we define transitions between states as changes in the adjacency matrices which form Markovian dynamics. The intense computational demand for deriving the transition probability matrices prompted us to develop *StreAM*-$T_g$, a stream-based algorithm for generating such Markov models of *k*-vertex adjacency matrices representing the RNA.

**Results:**

We benchmark *StreAM*-$T_g$ (a) for random and RNA unit sphere dynamic graphs (b) for the robustness of our method against different parameters. Moreover, we address a riboswitch design problem by applying *StreAM*-$T_g$ on six long term molecular dynamics simulation of a synthetic tetracycline dependent riboswitch (500 ns) in combination with five different antibiotics.

**Conclusions:**

The proposed algorithm performs well on large simulated as well as real world dynamic graphs. Additionally, *StreAM*-$T_g$ provides insights into nucleotide based RNA dynamics in comparison to conventional metrics like the root-mean square fluctuation. In the light of experimental data our results show important design opportunities for the riboswitch.

## Background

The computational design of switchable and catalytic ribonucleic acids (RNA) becomes a major challenge for synthetic biology [1]. So far, available models and simulation tools to design and analyze functionally complex RNA based devices are very limited [2]. Although several tools are available to assess secondary as well as tertiary RNA structure  [3], current capabilities to simulate dynamics are still underdeveloped [4] and rely heavily on atomistic molecular dynamics (MD) techniques [5]. RNA structure is largely modular and composed of repetitive motifs [4] that form structural elements such as hairpins and stems based on hydrogen-bonding patterns [6]. Such structural modules play an important role for nano design [1, 7].

In order to understand RNA dynamics [8, 14] we develop a new method to quantify all possible structural transitions, based on a coarse grained, transferable representation of different module sizes. The computation of Markov State Models (MSM) have recently become practical to reproduce long-time conformational dynamics of biomolecules using data from MD simulations [15].

To this end, we convert MD trajectories into dynamic graphs and derive the Markovian dynamics in the space of adjacency matrices. Aggregated matrices for each nucleotide represent RNA coarse grained dynamics. However, a full investigation of all transitions is computationally expensive.

To address this challenge we extend *StreaM*—a stream-based algorithm for counting 4-vertex motifs in dynamic graphs with an outstanding performance for analyzing (bio)molecular trajectories [16]. The extension *StreAM* computes one transition matrix for a single set of vertices or a full set for combinatorial many matrices. To gain insight into global folding and stability of an RNA molecule, we propose *StreAM*-$T_g$: It combines all adjacency-based Markov models for a nucleotide into one global weighted stochastic transition matrix $T_g(a)$. However, deriving Markovian dynamics from MD simulations of RNA is an emerging method to describe folding pathways [13] or to elucidate the kinetics of stacking interactions [11]. Especially MSM of atomistic aptamer simulations like the theophylline [12] and thrombin aptamer could help to understand structure-function relationships as well as the folding process [18]. Nonetheless, all the methods mentioned above rely on Root Mean Square Deviation (RMSD) computations in combination with clustering in order to identify relevant transition states. For *StreAM*-$T_g$, the transition states are given by small adjacency matrices representing structural motifs.

The remainder of this paper is structured as follows: In “Our approach for coarse grained analysis”, we introduce the concept of *StreAM*-$T_g$ as well as our biological test setup. We describe details of the algorithm in “Algorithm”. We present runtime evaluations as well as application scenario of our algorithm in “Evaluation” for a synthetic tetracycline (TC) dependent riboswitch (TC-Aptamer). Furthermore, we investigate the influence upon ligand binding of four different TC derivates and compare them with a conventional method. Finally, we summarize our work in “Summary, conclusion, and future work”.

## Our approach for coarse grained analysis

### Structural representation of RNA

Predicting the function of complex RNA molecules depends critically on understanding both, their structure as well as their conformational dynamics [17, 19]. To achieve the latter we propose a new coarse grained RNA representation. For our approach, we start with an MD simulation to obtain a trajectory of the RNA. We reduce these simulated trajectories to nucleotides represented by their ($C3'$) atoms. From there, we represent RNA structure as an undirected graph [20] using each $C3'$ as a vertex and distance dependent interactions as edges [3]. It is well known that nucleotide-based molecular interactions take place between more than one partner [21]. For this reason interactions exist for several edges observable in the adjacency matrix (obtained via a Euclidean distance cut-off) of $C3'$ coordinates at a given time-step. The resulting edges represent, e.g., strong local interactions such as Watson-Crick pairing, Hoogsteen, or $\pi{-}\pi$-stacking.

Our algorithm estimates adjacency matrix transition rates of a given set of vertices (nucleotides) and builds a Markov model. Moreover, by deriving all Markov models of all possible combinations of vertices, we can reduce them afterwards into a global weighted transition matrix for each vertex representing the ensemble that the nucleotide modeled as a vertex is immersed in.

### Dynamic graphs, their analysis, and Markovian dynamics

A *graph* $G = (V,E)$ is an ordered pair of *vertices* $V = \{v_1, v_2, \dots v_{|V|}\}$ and *edges* *E*. We refer to a single vertex of *V* as *a*. Here, we only consider *undirected graphs without self-loops*, i.e., $E \subseteq \{\{v,w\}: v,w \in V, v \ne w\}$. We define a self-loop as an edge that connects a vertex to itself. For a subset $V'$ of the vertex set *V*, we refer to $G[V'] = (V', E'), \; E' := \{\{v,w\} \in E : v, w \in V'\}$ as the $V'$ *-induced subgraph* of *G*. We refer to the powerset of *V* as $\mathbb {P}(V)$. The *adjacency matrix* $A(G) = A_{i,j}$ (Eq. 1) of a graph *G* is a $|V| \times |V|$ matrix, defined as follows:

1 $$\begin{aligned} A_{i,j} := \left\{ \begin{array}{rl} 0 &{} : i< j \wedge \{v_i, v_j\} \notin E \\ 1 &{} : i < j \wedge \{v_i, v_j\} \in E \\ \Diamond &{} : \text {otherwise} \end{array} \right. \end{aligned}$$

**Fig. 1:**
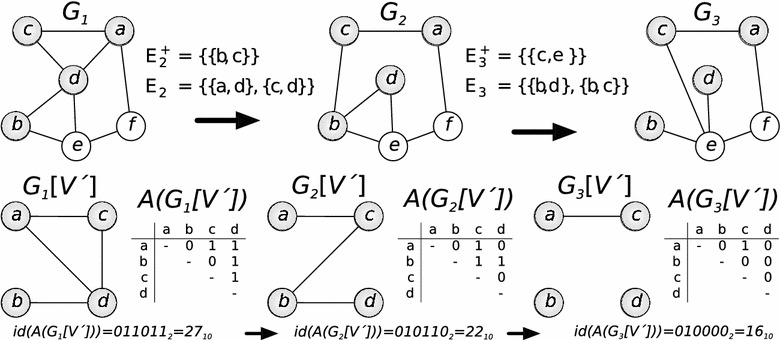
Dynamic graph example. Example of a dynamic graph and induced subgraphs for $V' = \{a, b, c, d\}$. The *first row* shows the dynamic graph $G_t$ and the second the induced subgraph $V'$ with its respective adjacency matrix. At the *bottom* is a short example of how to compute the adjacency id for the displayed subgraphs

Here, the symbol $\Diamond$ denotes for an undefined matrix entry. We denote the set of all adjacency matrices of size *k* as $\mathcal{A}_k$, with $|\mathcal{A}_k| = 2^{\frac{k \cdot (k-1)}{2}}$. In our current implementation *k* can takes values in $\{2,3,4,5,6,7,8,9,10\}$. With *concat*(*A*), we denote the row-by-row *concatenation* of all defined values of an adjacency matrix *A*. We define the *adjacency id* of a matrix *A* as the numerical value of the binary interpretation of its concatenation, i.e., $id(A) = concat(A)_2 \in \mathbb {N}$. We refer to $id(V') := id(A(G[V']))$ as the adjacency id of the $V'$-induced subgraph of *G*. For example, the concatenation of the adjacency matrix of graph $G_1[V']$ (shown in Fig. 1) is $concat(A(G_1[V'])) = \text {011011}$ and its adjacency id is $id(V') = 011011_2 = 27_{10}$.

As a *dynamic graph* $G_t = (V, E_t)$, we consider a graph whose edge set changes over time. For each point in time $t \in [1,\tau ]$, we consider $G_t$ as the *snapshot* or *state* of the dynamic graph at that time. The *transition of a dynamic graph* $G_{t-1}$ to the next state $G_{t}$ is described by a pair of edge sets which contain the edges added to and removed from $G_{t-1}$, i.e., $(E^+_{t}, E^-_{t})$. We refer to these changes as a *batch*, defined as follows: $E^+_{t} := E_{t} \backslash E_{t-1}$ and $E^-_{t} := E_{t-1} \backslash E_{t}$. The *batch size* is referred as $\delta _t=|E^+_t|+|E^-_t|$ and the average batch size is refered as $\delta _{avg}$ and is defined as $\frac{\sum _t \delta _t}{\tau }$.

The *analysis* of dynamic graphs is commonly performed using *stream-* or *batch-based* algorithms. Both output the desired result for each snapshot $G_t$. Stream-based algorithms take a single update to the graph as input, i.e., the addition or removal of an edge *e*. Batch-based algorithms take a pair $(E^+_{t+1},E^-_{t+1})$ as input. They can always be implemented by executing a stream-based algorithm for each edge addition $e \in E^+_{t+1}$ and removal $e \in E^-_{t+1}$. We refer to $id_t(V')$ as the adjacency id of the $V'$-induced subgraph of each snapshot of $G_t$. The result of analyzing the adjacency id of $V'$ for a dynamic graph $G_t$ is a list $(id_t(V'): t \in [1,\tau ])$. We consider each pair $(id_t(V'), id_{t+1}(V'))$ as an *adjacency transition of* $V'$ and denote the *set of all transitions* as $\mathcal{T}(V')$. Then, we define the *local transition matrix* $T(V')$ of $V'$ as a $|\mathcal{A}_k| \times |\mathcal{A}_k|$ matrix, which contains the number of transitions between any two adjacency ids over time, i.e., $T_{i,j}(V') := |(i+1,j+1) \in \mathcal{T}(V')|$ for an adjacency size *k*. From $T(V')$, we can derive a *Markov model* to describe these transitions.

By combining all possible $T(V')$ where $V' \in \mathbb {P}(V): |V'| = k$ and $a \in V'$, we derive a transition tensor $C_{a}(V)$. Thus $C_{a}(V)$ has the dimensions of $$|\mathcal{A}_k| \times |\mathcal{A}_k| \times (k-1)! \left( {\begin{array}{c}|V|\\ k-1\end{array}}\right)$$.

We define the weighting matrix $W(V')$ with the dimensions of $$|\mathcal{A}_k| \times (k-1)! \left( {\begin{array}{c}|V|\\ k-1\end{array}}\right)$$. $W(V')$ contains the weighting for every subset $V' \in C_{a}(V)$. It is defined as $W(V'):= \frac{S(V')}{\sum _{V' \in C_a(V)} S(V')}$. Here, $S(V')$ is a matrix containing the sum of every transition between adjacency $id(V')$ and every other $id(V')$ of the same matrix $T(V')$ for all $V' \in C_a(V)$. Hence $S(V')$ has the dimensions $$|\mathcal{A}_k| \times (k-1)! \left( {\begin{array}{c}|V|\\ k-1\end{array}}\right)$$. Thus $W(V')$ is considered as the local distribution weighted by its global distribution of transitions matrices of $V'$. Finaly, we define a global transition matrix, a vertex *a* is immeresd in, as $T_g(a)=\sum _{V' \in C_{a}(V)} W(V') \times T(V')$ with the dimensions $|\mathcal{A}_k| \times |\mathcal{A}_k|$.

For a local or global transition matrix the respective dominant eigenvector^1^ is called $\pi$ and represents the stationary distribution attained for infinite (or very long) times. The corresponding conformational entropy of the ensemble of motifs is $H:=-\sum _i \pi _i \cdot \log \pi _i$. The change in conformational entropy upon, e.g., binding a ligand is then given as $\Delta H = H_{wt}-H_{complex}$.

### MD simulation setup

**Fig. 2:**
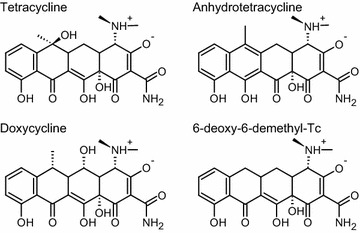
TC-derivates. TC-derivates illustrated as chemical structures. Here we show the structure of Tetracycline (*left top*), Anhydrotetracycline (*right top*), Doxycycline (*left bottom*) and 6-deoxy-6-demethyl-Tetracycline (*right bottom*). The illustrated derivates share the characteristic 4-ring-structure and functional groups

We use a structure of a synthetic tetracycline binding riboswitch (PDB: 3EGZ, chain B, resolution: 2.2 Å, Fig. 2) [23] and perform six simulations: the TC-Aptamer with five different tetracycline types in complex and one without tetracycline. As tetracycline binding alters the structural entropy of the molecule [24] our proposed method should be able to detect changes in (local) dynamics due the presence of tetracycline. All simulations were performed using the GROMACS software package (version 2016). For water molecules, we used the TIP3P model, the RNA interact through the CHARMM force field, while the tetracycline analogs interact through a modified CHARMM force field from Aleksandrov and Simonson [25, 26]. The systems were first energy minimized and equilibrated for 1 ns in the NVT-ensemble at a temperature of 300 K and for 5 ns in the NpT-ensemble at a temperature of 300 K and a pressure of 1 bar. During the equilibration, temperature was controlled using the velocity-rescale thermostat [27] ($\tau _{\text {T}} = {0.1}~{\mathrm{ps}}$) and pressure was controlled using the Berendsen barostat [28] ($\tau _{\text {P}}={0.5}~{\mathrm{ps}}$). Isothermal compressibility was set to ${4.5}\times 10^{-5}\,\mathrm{bar}^{-1}$, which is the corresponding value for water. Production runs were performed for 500 ns. The temperature was controlled using the Nosé-Hoover thermostat [29, 30] ($\tau _{\text {T}} = {1}~{\mathrm{ps}}$) and pressure was controlled using the Parrinello-Rahman barostat [31] ($\tau _{\text {P}}={1}~{\mathrm{ps}}$) during the production runs. Bond lengths were constrained using the LINCS [32] algorithm. The Lennard-Jones nonbonded interactions were evaluated using a cutoff distance of 1.2 nm. The electrostatic interactions were evaluated using the particle mesh Ewald method with a real space cutoff 1.2 nm and a grid-spacing 0.12 nm. Long-range corrections to energy and pressure due to the truncation of Lennard-Jones potential were accounted for. The equations of motion were integrated using a 2 fs time step.

#### Tetracycline derivates

**Fig. 3:**
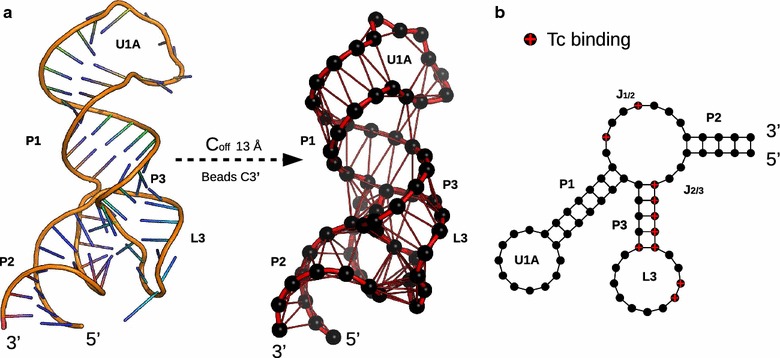
Structural representation of TC-Aptamer. **a** Crystal structure of TC-Aptamer with a cut-off of 13 Å and using $C3'$ atom for coarse graining reveals edges for dominant WC base-pairings. Important structural parts are annotated according to [23]. **b** Secondary structure representation of TC-Aptamer. Nucleotides are displayed as vertices and connections are based on hydrogen-bonding patterns. Nucleotides participating in TC-binding are colored in *red*. Graphics were created using Pymol and R [39, 47]

For the comparison of TC derivates we use tetracycline (tc), doxycycline (dc), anhydrotetracycline (atc) and 6-deoxy-6-demythyltetracycline (ddtc) in our MD simulation. These four analogs share the characteristic 4-ring-structure and functional groups of all tetracyclines. Still, the possibility and the mode of interaction with the RNA is an open question. The first ring of tetracycline carries a dimethylamino group, while the third ring carries a hydroxy and a methyl group facing towards the same direction away from the 4-ring-system. The detailed chemical structures are shown in Fig. 3. In comparison to these two rings the fourth, aromatic ring has an especially small steric volume on this side of the molecule. From tc over dc and atc to ddtc this steric volume is further reduced by shifting the aforementioned hydroxy and methyl group away from the fourth ring or eliminating some of them entirely. Note, that our graph-based approach is capable to easily distinguish between different modes of interaction upon changes in the, e.g., the side-chains of the rings. The molecular data of tc, dc, atc and ddtc was created using the Avogadro software [33]. Structures were manually constructed and moved into the extended conformation described to be 3 kcal/mol more stable than its twisted alternative by Alexandrov et al. [24]. The molecules were then fitted to the position of 7-chlorotetracycline (7-cl-tc) bound in the TC-Aptamer structure used for simulation. Note, that the geometry of 7-cl-tc was already present in the crystal structure of the TC-Aptamer. All considered antibiotics show different properties upon ligand binding. They range from high activity (tc, 7-cl-tc) to weak activity (dc, ddtc, atc) based on in vivo experiments [34].

### Workflow

#### RNA trajectory and contact probability

An RNA trajectory *X* is represented as a list of *T* frames $X = (\vec {x}_{t_0},\vec {x}_{t_1}, \ldots )$. Each frame $\vec {x}_t\in \mathbb {R}^{3n}$ contains the three-dimensional coordinates of the simulated system of the *n* atoms at the respective point in time *t*. We define a binary contact matrix *B*(*t*) with dimensions $|V| \times |V|$. Its entries scan range between $\{0,1\}$. A single contact $B_{i,j}(t)$ between one pair of atom coordinates $\vec {r}_{i}(t)$ and $\vec {r}_{j}(t)$ is generated if their Euclidean distance [L2-norm, $L2(\ldots )$] is shorter than *d*. Thus *B*(*t*) entries are defined as follows:

2 $$\begin{aligned} B_{i,j}(t) := \left\{ \begin{array}{rl} 0 &{} : d < L2(\vec {r}_{i}(t)-\vec {r}_{j}(t))\\ 1 &{} : d > L2(\vec {r}_{i}(t)-\vec {r}_{j}(t))\\ \end{array} \right. \end{aligned}$$

The contact probability of one pair of atom coordinates $\vec {r}_{i}$ and $\vec {r}_{j}$ is defined as:

3 $$\begin{aligned} P(X,\vec {r}_{i},\vec {r}_{j}) = \frac{\sum _{t=1}^T B_{ij}(t)}{T}. \end{aligned}$$

#### Graph transformation

All considered MD simulations have a total length of 500 ns using an integration stepsize of 2 fs. We created snapshots every 250 ps resulting in 100,000 frames. We generated dynamic graphs $G_t = (V, E_t)$ containing $|V|=65$ vertices (Table 1), each modelling a nucleic $3C'$ (Fig. 2). This resolution is sufficient to represent both small secondary structure elements as well as large quaternary RNA complexes [35, 36]. We create undirected edges between two vertices in case their Euclidean cut-off (*d*) is shorter than $\{ d \in N | 10 \le d \le 15 \}$ Å (cmp. Table 1).

#### Markov state models (MSM) of local adjacency and global transition matrix

*StreAM* counts adjacency transitions (e.g. as a set $\mathcal{T}(V')$) of an induced subgraph for a given adjacency size. Now the transition matrix $T(V')$ can be derived from $\mathcal{T}(V')$ but not all possible states are necessarily visited in a given, finite simulation, although a “missing state” potentially might occur in longer simulations. In order to allow for this, we introduce a minimal pseudo-count [37] of $P_k=\frac{1}{|\mathcal{A}_k|}$. All models that fullfill $\{V' \in \mathbb {P}(V) : |V'| = k, a \in V'\}$ have the same matrix dimension and thus can be envisioned to be combined in a tensor $C_a(V)$. Now, $C_{a~i,j,l}(V)$ is one entry of the tensor of transitions between adjacency *id* *i* and *j* in the *l* th transition matrix $T(V')$ with $$|l|=\left( {\begin{array}{c}|V|\\ k-1\end{array}}\right) \times k-1$$. Thus $C_a(V)$ contains all $T(V')$ a specific vertex is immersed in and due to this it contains all possible information of local markovian dynamics. To derive $T_g(a)$ every entry $C_{a~i,j,l}(V)$ is normalized by the count of all transitions of *i* in all matrices $S(V)_{j,l} = \sum _{i} C_{a~i,j,l}(V)$. For a given set of *l* transition matrices $T(V')$ we can combine them into a global model with respect to their probability:

4 $$\begin{aligned} T_{g~i,j}(a) = \sum _{l} \frac{S(V)_{jl}}{\sum _{l} S(V)_{jl}} \cdot C_{a~i,j,l}(V) . \end{aligned}$$

#### Stationary distribution and entropy

As $T_g(a)$ (Eq. 4) is a row stochastic matrix we can compute its dominant eigenvector from a spectral decomposition. It represents a basic quantity of interest: the stationary probability $\vec {\pi }:=\left( \pi _1, \ldots , \pi _{i},\ldots \right)$ of micro-states *i* [37]. To this end we used the markovchain library in R [38, 39]. For measuring the changes in conformational entropy $H := -\sum _{i=1}^{|\mathcal{A}_k|}{\pi _i \cdot \log \pi _i}$ upon binding a ligand, we define $\Delta H = H_{wt}-H_{complex}$, form a stationary distribution.

#### Conventional analysis: root mean square fluctuation (RMSF)

The flexibility of an atom can be quantitatively assessed by its *Root-mean-square fluctuation* (RMSF). This measure is the time average L2-norm $L2(\ldots )$ of one particular atom’s position $\vec {r}_{i}(t)$ to its time-averaged position $\bar{\vec {r}_{i}}$. The RMSF of an nucleotide *i* (represented by its respective $C3'$ atom) is defined as:

5 $$\begin{aligned} RMSF(X,r_{i}) := \sqrt{\frac{1}{T} \cdot \sum _{t=1}^T L2(\vec {r}_{i}(t),\bar{\vec {r}_{i}}~)^2} \end{aligned}$$

## Algorithm

### Overview

In this section, we introduce the required algorithms to compute $T_g(a)$. First, we describe *StreAM*, a stream-based algorithm for computing the adjacency $id(V')$ for a given $V'$. Afterwards we describe, the batch-based computation using *StreAM* $_B$ to derive $id_t(V')$. By computing the adjacency id of a dynamic graph $G_t[V']$ we derive a list $(id_t(V'): t \in [1,\tau ])$ where each pair $[id_t(V'), id_{t+1}(V')]$ represents an adjacency transition. The respective transitions are than stored in $\mathcal{T}(V')$. Now, a single $T(V')$ can be derived by counting the transitions in $\mathcal{T}(V')$. At last we introduce *StreAM*-$T_g$, an algorithm for the computation of a global transition matrix $T_g(a)$ for a given vertex *a* from a dynamic graph $G_t[V]$. To this end, *StreAM*-$T_g$ computes the tensor $C_a(V)$ which includes every single matrix $T(V')$ where $V' \in \mathbb {P}(V)$ and $|V'| = k$ with vertex $a \in V'$. Finally, *StreAM*-$T_g$ computes $T_g(a)$ from $C_a(V)$.

### StreAM and StreAM$_B$.

We compute the adjacency id $id(V')$ for vertices $V' \subseteq V$ in the dynamic graph $G_t$ using the stream-based algorithm *StreAM*, as described in Algorithm 1. Here, $id(V') \in [0,|\mathcal {A}_{|V'|}|)$ is the unique identifier of the adjacency matrix of the subgraph $G[V']$. Each change to $G_t$ consists of the edge $\{a,b\}$ and a type to mark it as addition or removal (abbreviated to *add*,*rem*). In addition to edge and type, *StreAM* takes as input the ordered list of vertices $V'$ and their current adjacency id.

An edge $\{a,b\}$ is only processed by *StreAM* in case both *a* and *b* are contained in $V'$. Otherwise, its addition or removal has clearly no impact on $id(V')$.

Assume $pos(V',a), pos(V',b) \in [1,k]$ to be the positions of vertices *a* and *b* in $V'$. Then, $i = min(pos(V',a), pos(V',b))$ and $j = max(pos(V',a), pos(V',b))$ are the row and column of adjacency matrix $A(G[V'])$ that represent the edge $\{a,b\}$. In the bit representation of its adjacency id $id(V')$, this edge is represented by the bit $(i-1) \cdot k + j - i \cdot (i+1)/2$. When interpreting this bit representation as a number, an addition or removal of the respective edge corresponds to the addition or subtraction of $2^{k \cdot (k-1) / 2 - ((i-1) \cdot k + j - i \cdot (i+1)/2)}$. This operation is performed to update $id(V')$ for each edge removal or addition. In the following, we refer to this position as $e(a,b,V') := \frac{|V'| \cdot (|V'|-1)}{2} - [(i-1) \cdot |V'| + j - \frac{i \cdot (i+1)}{2}]$.

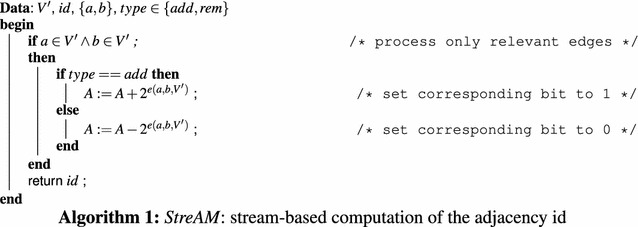



Furthermore, in Algorithm 2 we show *StreAM* $_B$ for the batch-based computation of the adjacency id for vertices $V'$

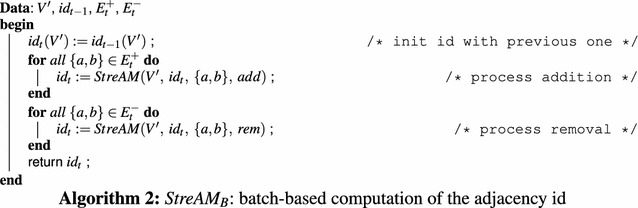



### StreAM-$T_g$

For the design or redesign of aptamers it is crucial to provide experimental researchers informations about e.g. dynamics at the nulceotide level. To this end, *StreAM*-$T_g$ combines every adajcency-based transition matrix, one nucleotide participates in, into a global model $T_g(a)$. This model can be derived for every nucleotide of the regarded RNA structure and contains all the structural transition of a nuclotide between the complete ensemble of remaining nucleotides. In order to do this, we present *StreAM*-$T_g$, an algorithm for the computation of global transition matrices, one particular vertex is participating in, given in Algorithm 3. A full computation with *StreAM*-$T_g$ can be divided into the following steps. The first step is the computation of all possible Markov models that fulfill $V' \in \mathbb {P}(V) : |V'| = k$ with *StreAM* for a given *k* with $k \in [2,10]$. This results in $$\left( {\begin{array}{c}|V|\\ k\end{array}}\right) \cdot k!=\frac{|V|!}{\left( |V|-k\right) !}$$ combinations. Afterwards, *StreAM*-$T_g$ sorts the matrices by vertex *id* into different sets, each with the size of $$\left( {\begin{array}{c}|V|\\ k-1\end{array}}\right) \cdot (k-1)!$$. For each vertex *a*, *StreAM*-$T_g$ combines the obtained $T(V')$ that fulfill $a \in V'$ in a transition tensor $C_a(V)$, which is normalized by $W(V')$ the global distribution of transition states a vertex is immersing in, taking the whole ensemble into account. $W(V')$ can be directly computed from $C_a(V)$ (e.g. “Dynamic graphs, their analysis, and Markovian dynamics”)

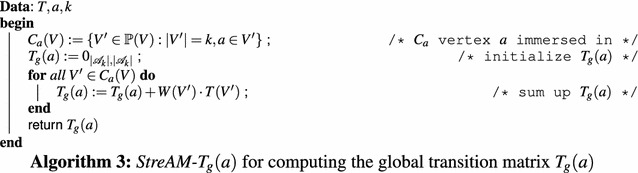



### StreAM-$T_g$ optimization using precomputed contact probability

The large computational demands for a full computation of the $$\left( {\begin{array}{c}|V|\\ k\end{array}}\right) \cdot k!=\frac{|V|!}{\left( |V|-k\right) !}$$ transition matrices to derive a set of $T_g(a)$, motivated us to implement an optimization: The number of Markov models can be reduced by considering only adjacencies including possible contacts between at least two vertices of $G_t = (V, E_t)$. This can be precomputed before the full computation by considering the contact probability $P(X,\vec {r}_{i},\vec {r}_{j})$ between vertices. To this end we only compute transition matrices forming a contact within the dynamic graph with $P(X,\vec {r}_{i},\vec {r}_{j}) > 0$.

## Evaluation

### Objectives

As *StreAM*-$T_g$ is intended to analyze large MD trajectories we first measure the speed of *StreAM* for computing a single $\mathcal{T}(V')$ to estimate overall computational resources. With this in mind, we benchmark different $G_t$ with increasing adjacency size *k* (Table 1). Furthermore, we need to quantify the dependence of computational speed with respect to $\delta _{t}$. Note, $\delta _{t}$ represents changes in conformations within $G_t$. For the full computation of $T_g(a)$, we want to measure computing time in order to benchmark *StreAM*-$T_g$ by increasing network size |*V*| and *k* for a given system due to exponentially increasing matrix dimensions $|\mathcal{A}_k| = 2^{\frac{k \cdot (k-1)}{2}}$ ($k=3$ 8, $k=4$ 64, $k=5$ 1,024, $k=6$ 32,768, $k=7$ 2,097,152 size of matrix dimensions). We expect due to combinatorial complexity of matrix computation a linear relation between |*V*| and speed and an exponential relation between increasing *k* and speed. To access robustness of influence of *d* robustness regarding the computation of $T_g(a)$ stationary distribution $\vec {\pi }$. We expect a strong linear correlation between derived stationary distributions. Details are shown in “Robustness against threshold”. We compare Markovian dynamics between the native TC-Aptamer and the structure in complex with 7-cl-tc with experimental data. We discuss the details in “Workflow” and “Application to molecular synthetic biology”. Furthermore, we want to illustrate the biological relevance by applying it to a riboswitch design problem; this is shown in detail in  “Application to molecular synthetic biology”. For the last part, we investigate the ligand binding of four different TC derivates using *StreAM*-$T_g$ and compare them with a classical metric (e.g. RMSF) in “Comparison of tetracycline derivates”.

### Evaluation setup

All benchmarks were performed on a machine with four *Intel(R) Xeon(R) CPU E5-2687W v2* processors with 3.4GHz running a Debian operating system. We implemented *StreAM* in Java; all sources are available in a GitHub repository.^2^ The final implementation *StreAM*-$T_g$ is integrated in a Julia repository.^3^ We created plots using the AssayToolbox library for R [39, 40]. We generate all random graphs using a generator for dynamic graphs^4^ derived for vertex combination.

**Table 1:** Details of the dynamic graphs obtained from MD simulation trajectories

	$\mathbf{10}$ **Å**	$\mathbf{11}$ **Å**	$\mathbf{12}$ **Å**	$\mathbf{13}$ **Å**	$\mathbf{14}$ **Å**	$\mathbf{15}$ **Å**	**Rand** $_{g1}$	**Rand** $_{g2}$	**Rand** $_{g3}$
|*V*|	65	65	65	65	65	65	500	500	500
|*E*|	94	129	189	241	298	353	500	1000	1200
$\delta _{avg}$	6.1	15.6	19.4	18	19.6	23.8	80	100	120

|*V*| is the number of vertices, |*E*| the number of edges and $$\delta _{t}$$ is the average batch size of a simulation. We convert simulations to unit sphere dynamic graphs with $$d \in [10, 15]$$ Å

#### Runtime dependencies of StreAM on adjacency size

For every dynamic graph $G_t(V,E_t)$, we selected a total number of 100,000 snapshots to measure *StreAM* runtime performance. In order to perform benchmarks with increasing *k*, we chose randomly nodes $k \in [3, 10]$ and repeated this 500 times for different numbers of snapshots (every 10,000 steps). We determined the slope (speed $\frac{frames}{ms}$) of compute time vs. *k* for random and MD graphs with different parameters (Table 1).

#### Runtime dependence of StreAM on batch size

We measured runtime performance of *StreAM* for the computation of a set of all transitions $\mathcal{T}(V')$ with different adjacency sizes *k* as well as dynamic networks with increasing batch sizes. To test *StreAM* batch size dependencies, 35 random graphs were drawn with increasing batch size and constant numbers of vertex and edges. All graphs contained 100,000 snapshots and *k* is calculated from 500 random combinations of vertices.

#### Runtime dependencies of StreAM-$T_g$ on network size

We benchmarked the full computation of $T_g(a)$ with different $k \in [3, 5]$ for increasing network sizes |*V*|. Therefore we performed a full computation with *StreAM*. *StreAM*-$T_g$ sorts the obtained transition list, converts them into transition matrices and combines them into a global Markov model for each vertex.

#### Runtime evaluation

**Fig. 4:**
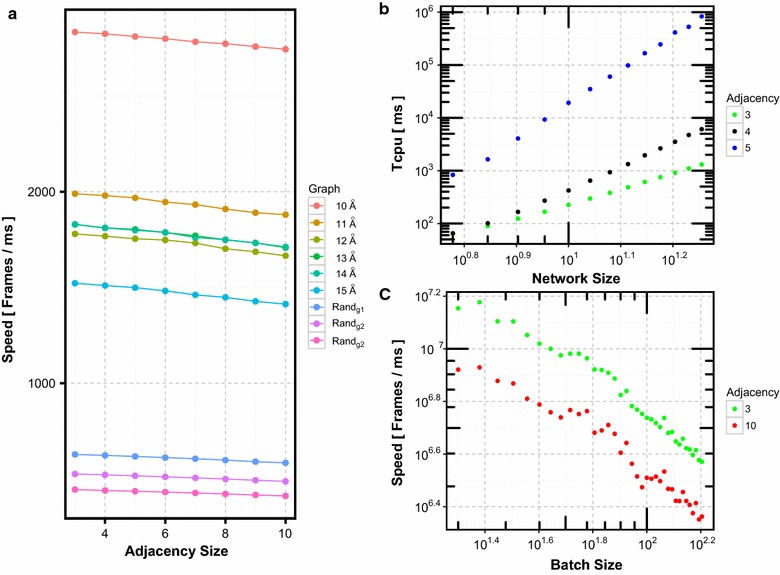
Runtime performance of StreAM-$T_g$. **a** Speed of computing a set of $\mathcal{T}(V')$ using *StreAM*. **b** Performance of $T_g(a)$ full computation with increasing network size |*V*| and different adjacency sizes $k=3,4,5$. **c** Speed of *StreAM* with increasing batch size for $k=3,10$

Figure 4b shows computational speeds for each dynamic graph. Speed decreases linearly with a small slope (Fig. 4a). While this is encouraging the computation of transition matrices for $k > 5$ is still prohibitively expensive due to the exponential increase of the matrix dimensions with $2^{\frac{k \cdot (k-1)}{2}}$. For $G_t$ obtained from MD simulations, we observe fast speeds due to small batch sizes (Table 1).

Figure 4b reveals that $T_{cpu}$ increases linearly with increasing |*V*| and with *k* exponentially. We restrict the $T_g(a)$ full computation to $k<5$. In Fig. 4c, speed decreases linearly with $\delta _{t}$. As $\delta _{t}$ represents the changes between snapshots our observation has implications for the choice of MD integration step lengths as well as trajectory granularity.

### Performance enhancing by precomputed contact probability

**Fig. 5:**
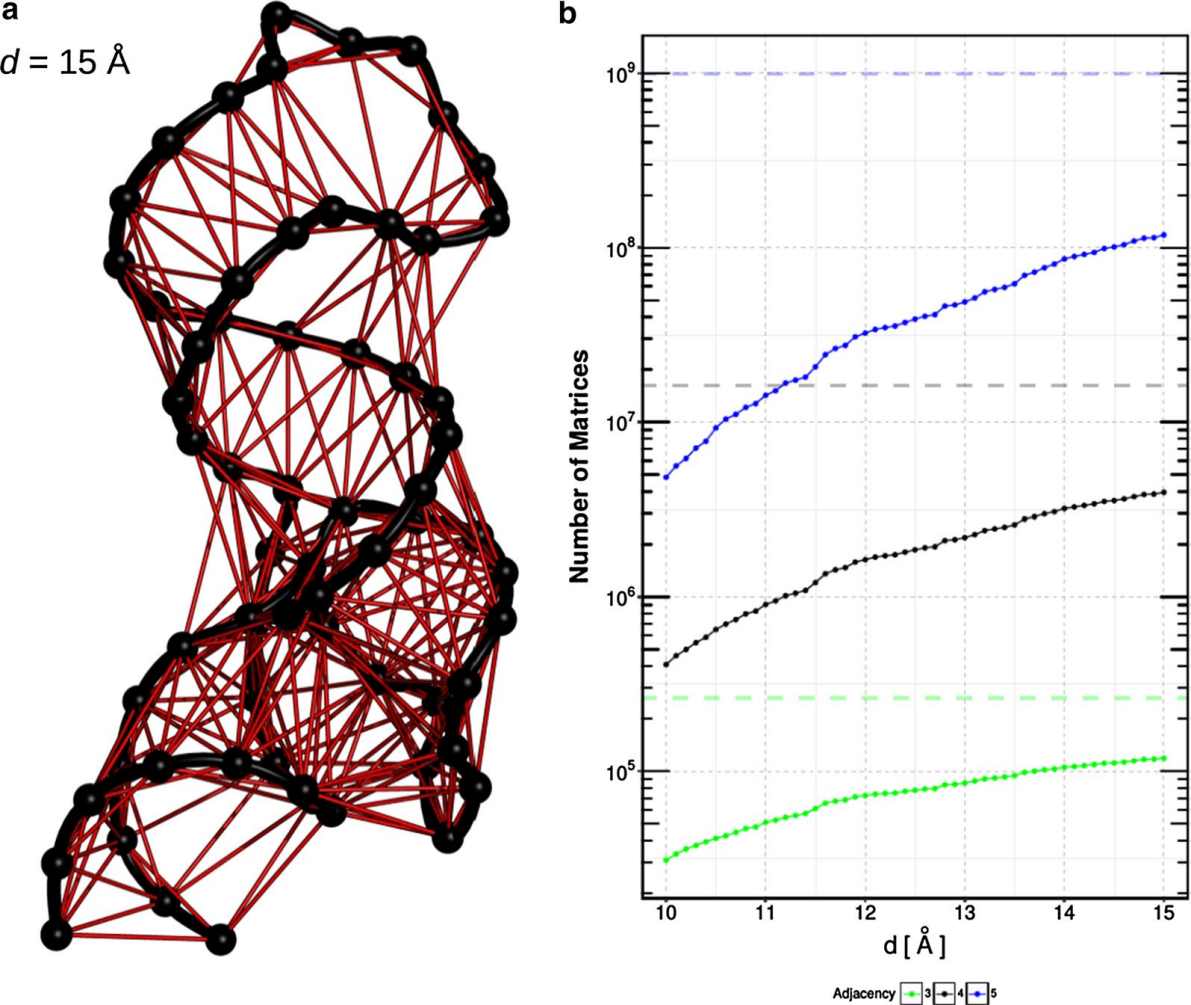
Precomputation with different cut-offs. **a** Illustration of the the first frame of the TC-Aptamer simulation without TC th created with a cut-off of $$d=15 $$ Å. Vertices (representing nucleotides) are colored in *black* and edges (representing interactions) in *red*. The edges belonging to the backbone are furthermore highlighted in *black*. Graphics were created using Pymol and R [39, 47]. **b** Number of $$\mathcal{T}(V')$$ for a full computation of $$T_g(a)$$ after selection with contact probability as function of cut-off *d* for three different adjacency sizes ($$k=3,4,5$$). The *dashed lines* show the number of matrices normally required for a full computation [$$k=3$$, 262,080 matrices (*green*); $$k=4$$ , 16,248,960 matrices (*black*); $$k=5$$, 991,186,560 matrices (*blue*)]

The exponential increase of transition matrix dimensions with $2^{\frac{k \cdot (k-1)}{2}}$ is an obvious disadvantage of the proposed method. However, there exist several $T(V')$ where every vertex is never in contact with another vertex from the set. These adjacencies remain only in one state during the whole simulation. To avoid the computation of the respective Markov models we precomputed $P(X,\vec {r}_{i},\vec {r}_{j})$ of all vertices. Thus only combinations are considered with $P(X,\vec {r}_{i},\vec {r}_{j}) > 0$. This procedure leads to a large reduction of $T_{cpu}$ due to fewer number of matrices to be computed to derive $T_g(a)$. To illustrate this reduction, we compute the number of adjacencies left after a precomputation of $P(X,\vec {r}_{i},\vec {r}_{j})$ as a function of *d* for the TC-Aptamer simulation without TC. The remaining number of transition matrices for adjacency sizes $k=3,4,5$ are shown in Fig. 5b. For further illustration we show the graph of the RNA molecule obtained for a cut-off of $d=15$ Å in Fig. 5a.

We can observe that using a precomputation of $P(X,\vec {r}_{i},\vec {r}_{j})$ to a full computation of $T_g(a)$ hardly depends on the Euclidean cut-off (*d*) for all considered adjacencies. The reduced computational costs in case of a full computation can be expressed by a significant smaller number of transition matrices left to compute for all considered adjacency sizes $k=3,4,5$. For example if we use $k=4$ and $d=13$ Å we have to compute 16,248,960 transition matrices, if we use a precomputation of $P(X,\vec {r}_{i},\vec {r}_{j})$ we can reduce this value to 2,063,100, this roughly eightfold. Furthermore, in case of new contact formation due to an increased *d* the number of transition matrices can increase.

### Robustness against threshold

Here, we investigate the influence of threshold *d* for the full computation of $T_g(a)$. To this end, we created dynamic graphs with different $d \in [11, 15]$ Å of the TC-Aptamer simulation without TC. Here, we focus on a simple model with an adjacency size of $k=3$, thus with eight states. In particular, we focus on the local adjacency matrix of combination 52, 54 and 51 because these nucleotides are important for TC binding and stabilization of intermediates.

**Fig. 6:**
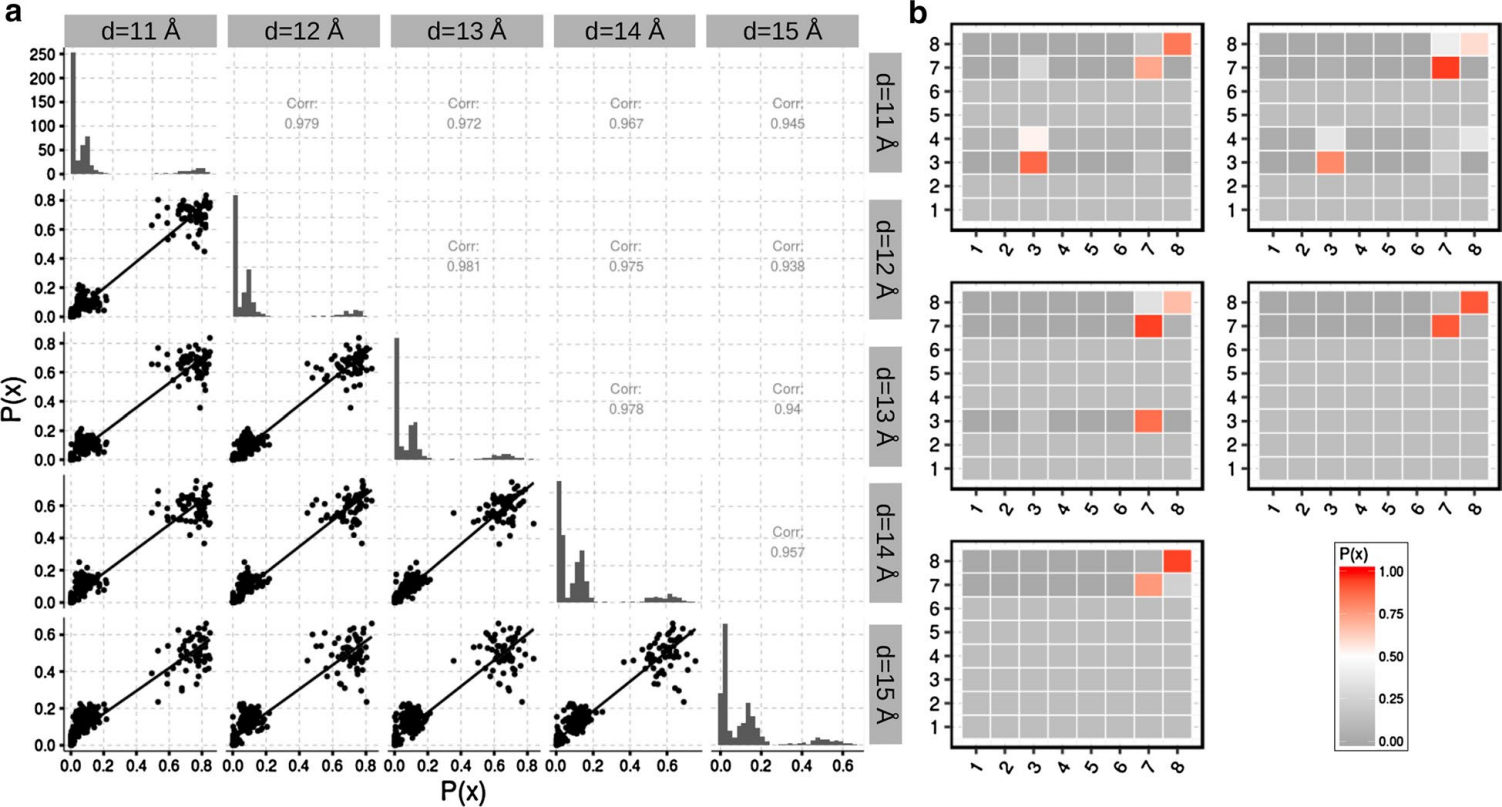
Robustness for $T_g(a)$ of the native riboswitch. **a** Scatter plot matrix of computed $\vec {\pi }$ for each $T_g(a)$ at different *d*. The* lower triangle* includes the scatterplots obtained at different *d*. The diagonal includes the histogram of all 65 $\vec {\pi }$ and the* upper triangle* includes the Pearson product moment correlation of the corresonding scatterplots. **b** Illustration of single $T(V')$ derived for vertex combination 52, 54 and 51 for $d \in [11, 15]$ Å as heat maps

To access the overall robustness of a full computation of $T_g(a)$ we compute the stationary distribution for every $T_g(a)$ and afterwards we compare them with each other. For the comparison we use the Pearson product moment correlation (Pearson’s *r*). Figure 6 illustrates the comparison of stationary distributions obtained from 65 $T_g(a)$ for unit sphere dynamic graphs with different *d*.

The obtained Pearson correlations *r* are also shown in Fig. 6 (a, upper triangle). We observed a high robustness expressed by an overall high correlation ($r= 0.938$ to $r = 0.98$) of the dynamic graphs created with different *d*. However transient states disappear with increasing threshold *d* (Fig. 6b). This observation stems from the fact that the obtained graph becomes more and more densely connected. One consequence of a high threshold *d* is that the adjacency remain in the same state.

### Accuracy of StreAM

In this section we discuss the accuracy of *StreAM* for the computation of a set of all transitions $\mathcal{T}(V')$ on finite data samples. Our approach estimates the transition probabilities from a trajectory as frequencies of occurrences. It could be shown that uncertainties derived from a transition matrix (e.g derived from a molecular dynamics simulation) decreases with increasing simulation time [22]. Thus the error and bias in our estimator are driven by the available data set size to derive $\mathcal{T}(V')$. Additionally, there is an implicit influence of *k* on the accuracy since the number of *k* determines the transition matrix dimensions. Consequently, the available trajectory (system) data must be at least larger than the number of entries in the transition matrix to be estimated in order to use *StreAM*.

### Application to molecular synthetic biology

This section is devoted to investigate possible changes in Markovian dynamics of the TC-Aptamer upon binding of 7-cl-tc. This particular antibiotic is part of the crystal structure of the TC-Aptamer thus structure of 7-cl-tc has the correct geometry and orientation of functional groups.

**Fig. 7:**
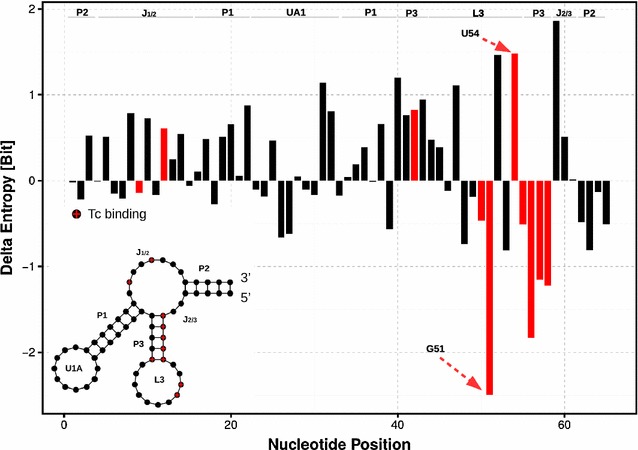
$\Delta H$ (in bit) comparison for 7-cl-tc. $\Delta H$ for $T_g(a)$ of the native riboswitch and the one in complex with 7-cl-tc. Nucleotides with 7-cl-tc in complex are colored in *red*. At the *top*, we annotate the nucleotides with secondary structure information. A positive value of $\Delta H$ indicates a loss and a negative a gain of conformational entropy

For both simulations of “Workflow”, we computed 16,248,960 transition matrices and combined them into 65 global models (one for each vertex of the riboswitch). To account for both the pair-interactions and potential stacking effects we focus on $k=4$-vertex adjacencies and use dynamic RNA graphs with $d=13$ Å. One global transition matrix contains all the transitions a single nucleotide participates in. The stationary distribution and the implied entropy (changes) help to understand the effects of ligand binding and potential improvements on this (the design problem at hand). The $\Delta H$ obtained are shown in Fig. 7.

A positive value of $\Delta H$ in Fig. 7 indicates a loss of conformational entropy upon ligand binding. Interestingly, the binding loop as well as complexing nucleotides gain entropy. This is due to the fact of rearrangements between the nucleotides in spatial proximity to the ligand because 70% of the accessible surface area of TC is buried within the binding pocket L3 [23]. Experiments confirmed that local rearrangement of the binding pocket are necessary to prevent a possible release of the ligand [41]. Furthermore crystallographic studies have revealed that the largest changes occur in L3 upon TC binding [23]. Furthermore, we observe the highest entropy difference for nucleotide G51. Experimental data reveals that G51 crosslinks to tetracycline when the complex is subjected to UV irradiation [42]. These findings suggest a strong interaction with TC and thus a dramatic, positive change in $\Delta H$. Nucleotides A52 and U54 show a positive entropy difference inside L3. Interestingly, molecular probing experiments show that G51, A52, and U54 of L3 are—in the absence of the antibiotic—the most modified nucleotides [23, 34]. Clearly, they change their conformational flexibility upon ligand binding due they direct interaction with the solvent. U54 further interacts with A51,A52,A53 and A55 building the core of the riboswitch [23]. Taken together, these observations reveal that U54 is necessary for the stabilization of L3. A more flexible dynamics ($\Delta H$) will change the configuration of the binding pocket and promotes TC release.

#### Comparison of tetracycline derivates

**Fig. 8:**
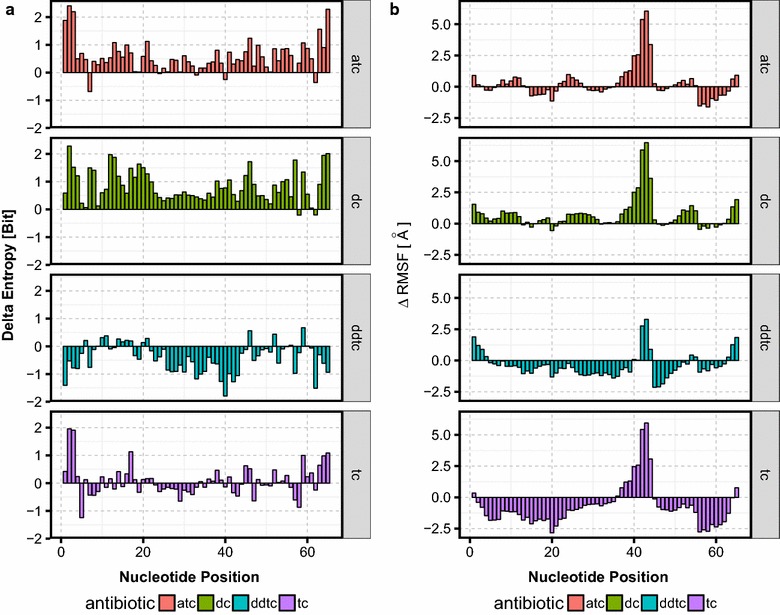
Comparison of $\Delta H$ and $\Delta RMSF$. **a** $\Delta H$ for $T_g(a)$ between the native riboswitch and the complex with four different TC derivates. $\Delta H$ is plotted against nucleotide position as a bar plot. A positive value of $\Delta H$ indicates a loss and a negative a gain of conformational entropy. **b** $\Delta RMSF$ between the native riboswitch and the complex with four different TC derivates (antibiotic). A positive value of $\Delta RMSF$ indicates a loss and a negative an increase in fluctuations

In this section, we want to investigate possible changes in configuration entropy by binding of different TC derivates. Moreover, we want to contrast *StreAM*-$T_g$ to conventional metrics like RMSF (Eq. 5) using the entropy of the stationary distributions obtained from $T_g(a)$. Therefore, we simulated a set consisting of four different antibiotics (atc, dc, ddtc, tc) in complex with the riboswitch of “Workflow”. The structures of all derivates, each with different functional groups and different chemical properties, are shown in Fig. 3. For this approach we use a precomputation of $P(X,\vec {r_{i}},\vec {r_{j}})$ to reduce the number of transition matrices for a full computation of $T_g(a)$. Hence for all four simulations of TC derivates, we computed 1,763,208 (for tc), 1,534,488 (for atc), 2,685,816 (for dc) and 2,699,280 (for ddtc) transition matrices and combined them into 65 global models $T_g(a)$ each. Similar to “Application to molecular synthetic biology”, we compute $\Delta H = H_{wt}-H_{complex}$ from the stationary distribution as well as $\Delta RMSF = RMSF_{wt}-RMSF_{complex}$ from individual RMSF computations. The results are shown in Fig. 8.

The $\Delta RMSF$ in Fig. 8b and in $\Delta H$ Fig. 8a shows a similar picture in terms of nucleotide dynamics. If we focus on atc we can observe a loss of conformational entropy upon ligand binding for almost every nucleotide. Considering this example the RMSF only detects a significant loss of nucleotide-based dynamics ranging from nucleotide 37–46. However, for dc, we observe the same effects like for dc. Contrary to this observation we detect, for ddtc, an increase in dynamic upon ligand binding as well as negative $\Delta RMSF$ values. For tc, we observe a similar picture as for 7-cl-tc (“Comparison of tetracycline derivates”). In a next step, we want to compare the obtained differences in stationary distribution with experimental values. To this end,we use an experimental metric: *xfold* values. A xfold value describes the efficiency of regulation in vivo and is given as the ratio of fluorescence without and with antibiotic in the experimental setup [43]. Unfortunately, atc reveals no experimental dynamics due to growth inhibition caused by the toxicity of the respective tc derivative [43]. In contrast to atc, dc and ddtc show only a weak performance (xfold = 1.1) in comparison to tc (xfold = 5.8) and 7-cl-tc (xfold = 3.8) [43]. On the one hand, atc and dc appear overall too rigid and on the other hand ddtc too flexible to obtain a stable bound structure, implying insufficient riboswitch performance. For our design criterion of high xfold, we conclude that only certain nucleotides are allowed to be affected upon ligand binding. In particular, we need flexible nucleotides for the process of induced ligand binding (like nucleotide G51 Fig. 7) and stabilization of the complex intermediates (“Application to molecular synthetic biology”). Additionally, the switch needs rigidity for nucleotides building the stem region of the TC-Aptamer upon ligand binding (like nucleotides A51, A52 and A53 Fig. 7).

## Summary, conclusion, and future work

Simulation tools to design and analyze functionally RNA based devices are nowadays very limited. In this study, we developed a new method *StreAM*-$T_g$ to analyze structural transitions, based on a coarse grained representation of RNA MD simulations, in order to gain insights into RNA dynamics. We demonstrate that *StreAM*-$T_g$ fulfills our demands for a method to extract the coarse-grained Markovian dynamics of motifs of a complex RNA molecule. Moreover *StreAM*-$T_g$ provides valuable insights into nucleotide based RNA dynamics in comparison to conventional metrics like the RMSF.

The effects observed in a designable riboswitch can be related to known experimental facts, such as conformational altering caused by ligand binding. Hence *StreAM*-$T_g$ derived Markov models in an abstract space of motif creation and destruction. This allows for the efficient analysis of large MD trajectories.

Thus we hope to elucidate molecular relaxation timescales, spectral analysis in relation to single-molecule studies, as well as transition path theory in the future. At present, we use it for the design of switchable synthetic RNA based circuits in living cells [2, 44].

To broaden the application areas of *StreAM*-$T_g$ we will extend it to proteins as well as evolutionary graphs mimicking the dynamics of molecular evolution in sequence space [45].

## Abbreviations

MD: molecular dynamics
RMSF: root-mean-square fluctuation
TC: tetracycline
dc: doxycycline
atc: anhydrotetracycline
ddtc: 6-deoxy-6-demythyltetracycline
7-cl-tc: 7-chlorotetracycline

## Footnotes

1 Guaranteed to exist due to the Perron-Frobenius theorem with an eigenvalue of $\lambda = 1$.

2
https://github.com/BenjaminSchiller/Stream.

3
http://www.cbs.tu-darmstadt.de/streAM-Tg.tar.gz.

4
https://github.com/BenjaminSchiller/DNA.datasets
